# Recent advances in managing differentiated thyroid cancer

**DOI:** 10.12688/f1000research.12811.1

**Published:** 2018-01-18

**Authors:** Livia Lamartina, Giorgio Grani, Cosimo Durante, Sebastiano Filetti

**Affiliations:** 1 Dipartimento di Medicina Interna e Specialità Mediche, Università di Roma “Sapienza”, Viale del Policlinico 155, 00161 Rome, Italy

**Keywords:** Thyroid cancer, radioisotopes, TMN staging

## Abstract

The main clinical challenge in the management of thyroid cancer is to avoid over-treatment and over-diagnosis in patients with lower-risk disease while promptly identifying those patients with more advanced or high-risk disease requiring aggressive treatment. In recent years, novel clinical and molecular data have emerged, allowing the development of new staging systems, predictive and prognostic tools, and treatment approaches. There has been a notable shift toward more conservative management of low- and intermediate-risk patients, characterized by less extensive surgery, more selective use of radioisotopes (for both diagnostic and therapeutic purposes), and less intensive follow-up. Furthermore, the histologic classification; tumor, node, and metastasis (TNM) staging; and American Thyroid Association risk stratification systems have been refined, and this has increased the number of patients in the low- and intermediate-risk categories. There is now a need for new, prospective data to clarify how these changing practices will impact long-term outcomes of patients with thyroid cancer, and new follow-up strategies and biomarkers are still under investigation. On the other hand, patients with more advanced or high-risk disease have a broader portfolio of options in terms of treatments and therapeutic agents, including multitarget tyrosine kinase inhibitors, more selective BRAF or MEK inhibitors, combination therapies, and immunotherapy.

## Introduction

The incidence of differentiated thyroid cancer (DTC) continues to rise worldwide, mostly because of the growing use of powerful diagnostic tools that permit the discovery of an increasing number of small papillary thyroid cancers (PTCs). Mortality from DTC, however, has changed minimally over the past five decades: the vast majority of patients with DTC have indolent tumors. The main clinical challenge is to avoid over-diagnosis in patients with low-risk disease or benign thyroid nodules while promptly identifying those patients with more advanced or high-risk tumors that require aggressive treatment approaches. In recent years, we have witnessed the emergence of new clinical and molecular data that have allowed the development of new staging systems, new predictive and prognostic tools, and new approaches to treatment. Here, we will review the main advances regarding the staging and management of DTC.

## Pathologic definition and staging systems

The fourth edition of the World Health Organization histologic classification of endocrine tumors, published in June 2017 ^1^, contains important revisions, which may have a broad impact on clinical practice. First, the follicular-derived neoplasms now include a new entity within the group of tumors with borderline histological features: the non-invasive follicular thyroid tumor with papillary nuclear features (NIFTP) ^2, 3^, an encapsulated follicular-variant PTC with no evidence of capsular or vascular invasion. NIFTPs may account for up to 20% of the tumors previously classified as PTCs. The word “cancer” has been eliminated from the definition of NIFTP to underline its excellent prognosis: no adverse outcomes (such as cancer-related death, distant or regional metastases, and structural or biochemical recurrence) have been recorded for these tumors. The explicitly stated objective is to reduce the intensity of treatment and follow-up and the psychological consequences of the diagnosis of cancer. This entity was first proposed by an international panel of pathologists and other professionals and has been endorsed by the American Thyroid Association (ATA). However, the seemingly excellent outcomes still need to be confirmed in long-term prospective studies. The current evidence is retrospective and of only moderate quality ^2^. Other relevant changes include the identification of 15 PTC variants and the distinction of follicular thyroid cancers (FTCs) into three subgroups (minimally invasive FTCs with capsular invasion only, encapsulated angioinvasive FTCs, and widely invasive FTCs), which reflect the prognostic relevance of vascular invasion. Given its unique molecular profile ^4^, the oncocytic variant is now considered a separate entity and is referred to as Hürthle cell carcinoma. Poorly differentiated thyroid cancer is also a separate entity, in accordance with the Turin criteria ^5, 6^.

The American Joint Cancer Committee/Union Internationale Contre le Cancer TNM staging system for thyroid cancer takes into account patient age, the size and extent of the primary thyroid tumor (T), lymph node involvement (N), and the presence of distant metastases (M). This system is aimed at predicting mortality (not recurrences). The eighth edition of this staging system contains important modifications. The age cutoff was raised from 45 to 55 years ^7^, the presence of minimal extrathyroidal extension is no longer relevant for the T classification, and the tumor stages have been redistributed ( Table 1 and Table 2). Regional lymph node metastases or the presence of gross extrathyroidal extension limited to strap muscle no longer mandate stage III but stage II. The presence of macroscopic invasion beyond strap muscle (subcutaneous tissue, larynx, trachea, esophagus in T4a tumors or carotid artery, prevertebral fascia or mediastinal vessels in T4b tumors) is an unfavorable prognostic factor. The overall goal of these modifications is to improve the accuracy of the system’s prediction of mortality ^8^, restricting the assignment of higher risk to a small subset of patients (5–10%) with tumors classified as stage III (pT4a, any N, M0) or stage IV (pT4b, any N, M0, and M1). Implementation of the eighth edition revisions is expected to result in the down-staging of approximately 30–40% of patients with thyroid cancer ^9, 10^.

**Table 1.** AJCC TNM seventh and eighth edition: definitions.

Seventh edition	Eighth edition
**Tumor**	
**T1a:** tumor ≤1 cm limited to the thyroid	**T1a:** tumor ≤1 cm limited to the thyroid
**T1b:** tumor >1 cm but ≤2 cm limited to the thyroid	**T1b:** tumor >1 cm but ≤2 cm limited to the thyroid
**T2:** tumor >2 cm but ≤4 cm limited to the thyroid	**T2:** tumor >2 cm but ≤4 cm limited to the thyroid
**T3:** tumor >4 cm limited to the thyroid or minimal extrathyroid extension (for example, perithyroidal soft tissues or sternothyroid muscle) from a tumor of any size	**T3a:** tumor >4 cm limited to the thyroid **T3b:** gross extrathyroidal extension invading only strap muscles (sternohyoid, sternothyroid, thyrohyoid, omohyoid) from a tumor of any size
**T4a:** gross extrathyroidal extension invading subcutaneous soft tissues, larynx, trachea, esophagus, or recurrent laryngeal nerve from a tumor of any size	**T4a:** gross extrathyroidal extension invading subcutaneous soft tissues, larynx, trachea, esophagus, or recurrent laryngeal nerve from a tumor of any size
**T4b:** gross extrathyroidal extension invading prevertebral fascia or encasing the carotid artery or mediastinal vessels from a tumor of any size	**T4b:** gross extrathyroidal extension invading prevertebral fascia or encasing the carotid artery or mediastinal vessels from a tumor of any size
**Node**	
**Nx:** regional lymph nodes cannot be assessed	**Nx:** regional lymph nodes cannot be assessed
**N0:** no evidence of locoregional lymph node metastasis	**N0a:** one or more cytologically or histologically confirmed benign lymph nodes **N0b:** no radiologic or clinical evidence of locoregional lymph node metastasis
**N1a:** ipsilateral or bilateral metastasis to level VI (pretracheal, paratracheal, or prelaryngeal/Delphian) lymph nodes	**N1a:** ipsilateral or bilateral metastasis to level VI or VII (pretracheal, paratracheal, or prelaryngeal/Delphian, or upper mediastinal) lymph nodes; this can be unilateral or bilateral disease
**N1b:** metastasis to unilateral, bilateral, or contralateral lateral neck lymph nodes (levels I, II, III, IV, or V) or retropharyngeal or superior mediastinal lymph nodes (level VII)	**N1b:** metastasis to unilateral, bilateral, or contralateral lateral neck lymph nodes (levels I, II, III, IV, or V) or retropharyngeal lymph nodes
**Metastasis**	
**M0:** no distant metastasis	**M0:** no distant metastasis
**M1:** distant metastasis	**M1:** distant metastasis

AJCC, American Joint Cancer Committee; TNM, tumor, node, and metastasis.

**Table 2.** AJCC TNM seventh and eighth edition: stage.

Seventh edition	Age <45 years			Eighth edition	Age <55 years		
I	Any T	Any N	M0	I	Any T	Any N	M0
II	Any T	Any N	M1	II	Any T	Any N	M1
**Seventh edition**	**Age ≥45 years**			**Eighth edition**	**Age ≥55 years**		
I	T1a/b	N0	M0	I	T1a/b T2	N0/NX N0/NX	M0 M0
II	T2	N0	M0	II	T1a/b T2 T3a/b	N1a/b N1a/b Any N	M0 M0 M0
III	T1a/b T2 T3	N1a N1a N0, N1a	M0 M0 M0	III	T4a	Any N	M0
IVa	T1a/b T2 T3 T4a	N1b N1b N1b N0, N1a, N1b	M0 M0 M0 M0	IVa	T4b	Any N	M0
IVb	T4b	Any N	M0	IVb	Any T	Any N	M1
IVc	Any T	Any T	M1	-	-	-	-

AJCC, American Joint Cancer Committee; TNM, tumor, node, and metastasis.

In the 2015 edition of its practice guidelines, the ATA also revised its system for stratifying the risk of recurrent disease. The new variables considered include new pathologic features of the tumor (for example, histopathological variant, vascular invasion, number of metastatic lymph nodes, size of the largest metastatic lymph node, and the presence of extranodal extension) as well as its molecular characteristics (mutational status of *BRAF* and the *TERT* promoter ^11^, when available) ^12^. These changes are expected to allow more precise estimates of the likelihood of recurrence.

The dynamic risk classification process used during follow-up assigns patients to one of four subgroups and may be modified at each follow-up examination: responses to therapy are classified as excellent, biochemically incomplete, structurally incomplete, or indeterminate response.

## Management strategies

Current international guidelines advocate personalized decision-making—based on the risk of recurrence and disease-specific death—regarding the extent of surgery, the use of radioactive iodine (RAI) therapy, the intensity and length of follow-up, and the degree of thyroid-stimulating hormone (TSH) suppression.

### Active surveillance

The 2015 ATA guidelines include active surveillance among the management options for small subcentimeter PTCs. In pivotal Japanese studies, this strategy appeared to be both safe and effective ^13, 14^: after 10 years, very few patients had experienced tumor growth (8%), and the development of lymph node metastases was even less common (4%). Age below 40 at diagnosis was an independent risk factor for disease progression ^15^. In terms of cures, delayed surgical treatment of these tumors was as effective as immediate treatment ^15^.

In a study conducted in the United States, 291 patients with cytologically suspicious or malignant thyroid nodules (Bethesda class V or VI) measuring 1.5 cm or less were managed with active surveillance for a median of two years ^16^. The percentages of tumors displaying growth were 2.5% at two years and 12% at five years. Independent predictors of growth were age under 50 years and clinical judgment as “inappropriate for active surveillance” ^16^. The latter label may be applied on the basis of nodule-related features (subcapsular location adjacent to the recurrent laryngeal nerve [RLN], suspicion of extrathyroidal extension, and invasion of the RLN or trachea—all three of which can be difficult to exclude on neck ultrasound [US]—fine-needle aspiration [FNA] cytology findings suggestive of an aggressive histotype, and a documented increase in size of at least 3 mm in a confirmed PTC) or patient-related factors (metastatic disease, age below 18 years, refusal of the surveillance-alone approach, poor adherence to the follow-up protocol) or physician-related factors (limited experience with thyroid cancer management or neck US or both) or a combination of these factors ^17^.

Other observational clinical trials to evaluate the “active surveillance” approach in subcentimeter PTCs are underway in Korea and Israel (NCT02952612, NCT02938702, and NCT02609685). Also, there is a need for biomarkers that can identify those rare microcarcinomas that are likely to grow, so they can be promptly referred for surgery.

### Individualized surgical approaches

According to the ATA guidelines ^12^, thyroid lobectomy (TL) may be used for low-risk, intrathyroidal tumors up to 4 cm in size with no lesions in the contralateral lobe. Total thyroidectomy (TT) was previously considered the preferred approach for these tumors. In a retrospective analysis of 52,173 cases in the Surveillance Epidemiology and End Results (SEER) database, TL for tumors measuring at least 1 cm was associated with small but statistically significant increases in the risks for recurrence (9.8% versus 7.7%) and mortality (2.9% versus 1.6%) compared with TT ^18^. A recent retrospective analysis with a more extensive risk stratification found no such difference in terms of overall survival ^19^, but, in another meta-analysis, the risk of recurrence after TL was significantly higher than that after TT (8.3% versus 4.4%; *p*<0.01) ^20^. It is worth noting that tumor recurrence in the contralateral lobe has been observed in 5% of patients treated with TL ^21^ and that benign nodule relapse is reported in 20–50% ^22, 23^. Levothyroxine treatment has been reported to prevent benign nodule relapse, but the evidence for this effect is limited ^24^.

TL offers several advantages over TT. First, the rate of side effects is lower with TL. It virtually eliminates the risks of permanent hypoparathyroidism and bilateral RLN palsy ^25^ and reduces the rates of permanent unilateral RLN palsy (0.6% in TL versus 1.3% in TT) ^25^. Second, surgical hypothyroidism after TT requires lifelong levothyroxine (LT4) replacement therapy. The rate of hypothyroidism after TL varies from 23.6 to 47% ^26, 27^, and patients with a normal serum TSH level may not require LT4 treatment at all. TL and TT are also believed to have differential effects on the patient’s quality of life. However, two relatively small studies have failed to detect any difference ^28, 29^ and thus further investigation of this issue is needed.

The ATA guidelines do not advocate prophylactic central neck dissection for low-risk patients ^12^ despite the high frequency of subclinical lymph node metastasis in DTC ^30^. In fact, microscopic lymph node metastases that are not clinically detected before surgery have a questionable role in patient outcome ^31^, and central compartment neck dissection carries an increased rate of surgical complications, hypoparathyroidism in particular ^32, 33^. In a prospective study of patients with no preoperative evidence of lymph node metastasis who were randomly assigned to undergo TT alone or TT with central compartment neck dissection, no difference in outcomes was found after five years of follow-up ^32^.

## Radioiodine remnant ablation: selective use

In the past, routine use of RAI ablation therapy after surgery was justified first by the need to eliminate residual normal thyroid tissue, to achieve an undetectable serum thyroglobulin (Tg) level. It also allowed the identification of persistent neoplastic tissue with a ^131^I whole-body scan (WBS) and was likely to destroy any occult nests of neoplastic cells, thereby improving long-term outcomes. These indications have been questioned in recent years ^12, 34, 35^. The ATA guidelines now recommend selective use of RAI based on individual risk ^12^. Claiming uncertainties and ambiguities in the evidence, the European Association of Nuclear Medicine refused to endorse these recommendations ^36^ and noted that there are no prospective, controlled study data that allow us to identify the patients with low-risk DTC who may not benefit from RAI ablation. However, the Association did not emphasize that a treatment should be given only in patients in whom it may be beneficial and did not acknowledge that uncertainties persist concerning benefits of RAI administration in low- and intermediate-risk patients.

It is generally agreed that RAI has no role in the management of patients with intra-thyroidal microcarcinomas. In other low- and intermediate-risk patients, the decision to ablate can be based on individual prognostic factors and on the serum Tg level measured 6 weeks after surgery either on LT4 treatment with a sensitive assay or following recombinant human TSH (rhTSH) injections. An undetectable or a low serum Tg level at that time supports a decision to avoid RAI administration. When it is indicated, it should consist of the administration of 1.1 GBq following rhTSH injections ^37^.

Two randomized clinical trials in Europe are enrolling low-risk patients and aim to obtain reliable data on the indications for post-operative RAI administration. In the ESTIMABL2 trial (NCT01837745) being conducted in France, 750 patients with a T1bN0,Nx tumor will be randomly assigned to post-operative ablation with an activity of 30 mCi after rhTSH stimulation or simple follow-up. The IoN trial in the UK (NCT01398085) has a similar design. The primary outcomes in the two studies are disease-free survival rates at 3 and 5 years, respectively.

## Follow-up tools

The main tools used for the follow-up of DTC are neck US and serum Tg determination ^38^. Undetectable serum Tg levels can reliably identify disease-free patients and have a negative predictive value close to 100%. In contrast, early minimally detectable levels have a low positive predictive value: the majority of patients with these findings remain free of structural disease during prolonged follow-up ^39^. Tg trends over time—instead of absolute values—should be monitored: declining levels are reassuring, whereas increases suggest the presence of growing thyroid tissue (normal or neoplastic) ^40^. In the presence of Tg autoantibodies (TgAbs), serum Tg levels determined by immunometric assays may be falsely low. In these cases, management can be guided by the temporal trends in the TgAb titers themselves ^41^. Novel biomarkers are emerging as replacements for serum Tg in these difficult cases, such as circulating microRNAs and other nucleic acids, but still need to be standardized and clinically validated ^42– 44^. The use of mass spectrometry for measuring serum Tg levels in the presence of TgAb also needs to be validated ^45, 46^. Sensitive Tg assays that can detect serum concentrations as low as 0.1 ng/mL are currently used and provide similar information on disease status as rhTSH-stimulated Tg obtained in the past with assays that had a sensitivity of 1 ng/mL. RhTSH-stimulated Tg levels are now measured during the follow-up only in those few patients with low but detectable serum Tg on LT4 treatment ^47^. In these patients, a substantial increase in the stimulated Tg level may indicate the presence of neoplastic tissue.

Neck US provides useful information. PTC almost always spreads first to the cervical lymph nodes, where it can be identified sonographically using specific criteria ^48^, thus eliminating the need for diagnostic ^131^I WBS. US is more cost-effective, eliminates radiation exposure, and has no adverse effects. Surgical treatment of lymph node metastases is recommended for lesions with smaller diameters exceeding 10 mm (for lateral N1) or 8 mm (for central N1), and there is no need for discovering small N1 of only a few millimeters in diameter. However, US is notoriously operator-dependent, and some findings are non-specific and classified as indeterminate. Lesions with such features display significantly lower rates of persistence and growth than those with more suspicious US characteristics ^49^. Suspicious findings can be confirmed by US-guided FNA with cytologic assessment and assay of Tg in the needle-washout fluid ^50^. Distant metastases are rare in patients with negative findings on neck US. However, in the presence of rising Tg levels or suspicious clinical features, second-line functional (diagnostic ^131^I WBS and 18-fluorodeoxyglucose positron emission tomography scan) or cross-sectional (computed tomography or magnetic resonance imaging) imaging studies may be performed ^51^.

Risk assessment is a dynamic process: the response to therapy is re-assessed on the basis of findings at each follow-up visit and expressed as excellent, indeterminate, biochemically incomplete, or structurally incomplete ^12, 39, 52^. During long-term follow-up, even patients who initially displayed a high risk of persistent or recurrent disease can be re-classified as having lower-risk disease, and their follow-up program can be less intensive than originally planned. Lifelong surveillance is still recommended ^12^. However, over 75% of recurrent lesions are identified within the first five years of follow-up, and late recurrence is very unlikely in low- or intermediate-risk patients with excellent responses to treatment ^53^.

TSH stimulates the proliferation of normal and neoplastic thyrocytes ^54^, and levothyroxine treatment significantly reduces DTC recurrence and cancer-related mortality ^55^. The optimal TSH level is unclear. TSH suppression increases the risk for atrial fibrillation and osteoporosis in older patients and the risk of angina in patients with ischemic heart disease ^56^. Suppressive therapy should take into account both the likelihood of complications and the risk of increasing tumor cell proliferation. For this reason, levothyroxine treatment is no longer recommended for low- and intermediate-risk patients with no evidence of disease. The goal in these cases is a serum TSH level within the normal range. Suppressive therapy is advocated only in patients with structural disease and no contraindications ^12^.

## Treatment of distant metastases

RAI ( ^131^I) is currently the first-line treatment for distant metastases that are RAI-avid. Traditionally, thyroid hormone withdrawal has been the preferred method of preparation for this type of patient because it is associated with higher neoplastic tissue uptake and slower clearance of RAI than that achieved with rhTSH preparation. Observational data have suggested that rhTSH preparation may also be effective in terms of response to treatment in these patients ^57^, but the evidence in support of this conclusion is currently insufficient to recommend rhTSH use in patients with metastatic DTC ^12^. The activity of RAI for the treatment of distant metastases can be calculated dosimetrically or a fixed empiric dose can be used. A large retrospective study of DTC patients with distant metastases found similar overall survival with the two approaches ^58^ after adjustments for age and tumor burden ^59^.

## Radioactive iodine-refractory disease

RAI-refractory DTC is defined as persistent neoplastic tissue that does not take up RAI; disease characterized by heterogeneous RAI uptake (that is, some lesions are RAI-avid and others are not); or disease that progresses after RAI treatment despite RAI uptake ^12^. Specific molecular profiles are more likely to result in RAI-refractory disease ^60, 61^. Even in the presence of distant metastases, most patients have asymptomatic, slowly progressive disease. RAI-refractory patients should benefit from local treatments (surgery, external beam radiotherapy, or thermal ablation, depending on the site of the lesion and local expertise) if they have symptoms or a high risk of local complications.

When disease progression occurs at multiple sites in patients with target lesions of more than 1–2 cm in diameter, treatment with tyrosine kinase inhibitors (TKIs) should be considered.

Two multitarget TKIs have been approved for the treatment of RAI-refractory DTC in the United States and Europe: sorafenib and lenvatinib ^62, 63^. These drugs have been shown to prolong the progression-free survival of patients with progressive RAI-refractory DTC, as compared with placebo (10.8 versus 5.8 months, hazard ratio [HR] 0.59 for sorafenib; 18.3 versus 3.6 months, HR 0.21 for lenvatinib), and objective response rates were 12% and 65%, respectively ^62, 63^. However, multitarget TKIs have side effects ^64^. Greater experience in their use and better knowledge of the risk factors for these adverse effects ^65, 66^ are likely to improve their tolerance.

For BRAF- or ALK-mutant tumors, specific inhibitors may be used ^64, 67^. Treatments capable of restoring RAI uptake have attracted great interest. Selumetinib (a selective MEK inhibitor) and dabrafenib (a selective BRAF inhibitor used in BRAF-mutated tumors) have been shown to increase RAI uptake by RAI-refractory tumor tissues ^68, 69^, and these encouraging results are now under investigation (NCT02393690 and NCT03244956). Immunotherapy also appears to be a promising approach to thyroid cancer, alone or in association with other drugs ^70^ (NCT02390739 and NCT03181100). These agents have yet to be approved by regulatory agencies, such as the U.S. Food and Drug Administration and the European Medicines Agency. Enrollment in clinical trials for patients with progressive metastatic disease should be considered and encouraged in order to improve both clinical case outcomes and medical knowledge in the field ^71^.

## Conclusions

In recent years, DTC treatment has become considerably more conservative, with less extensive surgery (or no surgery at all), reduced use of radioisotopes, and less intensive follow-up of low- and intermediate-risk patients. Furthermore, the systems used to histologically classify and stage DTCs have recently been refined, along with the ATA scheme for estimating their risk of recurrence, and more patients are now considered to be at low or intermediate risk. There is a need for new, prospective data to clarify how these changing practices will impact the long-term outcome of these patients. On the other hand, patients with more advanced or high-risk disease now have a broader portfolio of treatment options, including multitarget TKI therapy, more selective BRAF inhibitors, combination therapies, and immunotherapy. However, the indications for each, their optimal starting times and dosing schedules, and their long-term safety profiles remain to be clarified in coming years.

## Abbreviations

ATA, American Thyroid Association; DTC, differentiated thyroid carcinoma; FNA, fine-needle aspiration; FTC, follicular thyroid cancer; HR, hazard ratio; NIFTP, non-invasive follicular thyroid neoplasm with papillary nuclear features; PTC, papillary thyroid carcinoma; RAI, radioactive iodine; rhTSH, recombinant human thyroid-stimulating hormone; RLN, recurrent laryngeal nerve; Tg, thyroglobulin; TgAb, thyroglobulin autoantibody; TKI, tyrosine kinase inhibitor; TL, thyroid lobectomy; TNM, tumor, node, and metastasis; TSH, thyroid-stimulating hormone; TT, total thyroidectomy; US, ultrasound; WBS, whole-body scan.
